# Trends in surgical techniques for the treatment of esophageal and gastroesophageal junction cancer: the 2022 update

**DOI:** 10.1093/dote/doac099

**Published:** 2023-01-12

**Authors:** E M de Groot, L Goense, B F Kingma, L Haverkamp, J P Ruurda, R van Hillegersberg

**Affiliations:** Department of Surgery, University Medical Center Utrecht, Utrecht, The Netherlands; Department of Surgery, University Medical Center Utrecht, Utrecht, The Netherlands; Department of Surgery, University Medical Center Utrecht, Utrecht, The Netherlands; Department of Surgery, University Medical Center Utrecht, Utrecht, The Netherlands; Department of Surgery, University Medical Center Utrecht, Utrecht, The Netherlands; Department of Surgery, University Medical Center Utrecht, Utrecht, The Netherlands

**Keywords:** esophageal cancer, esophagectomy, survey, worldwide practice

## Abstract

The aim of this study was to evaluate the current practice in surgical techniques for esophageal and gastroesophageal junction cancer surgery worldwide and to compare the results to the previous surveys in 2007 and 2014. An online survey was sent out among surgical members of the International Society for Diseases of the Esophagus, the World Organization for Specialized Studies on Disease of the Esophagus, the International Gastric Cancer Association, the Association of Upper Gastrointestinal Surgery of Great Britain and Ireland and Dutch gastroesophageal surgeons via the network of the investigators. In total, 260 surgeons completed the survey representing 52 countries and 6 continents; Europe 56%, Oceania 14%, Asia 14%, South-America 9%, North-America 7%. Of the responding surgeons, 39% worked in a hospital that performed >51 esophagectomies per year. Total minimally invasive esophagectomy was the preferred technique (53%) followed by hybrid esophagectomy (26%) of which 7% consisted of a minimally invasive thoracic phase and 19% of a minimally invasive abdominal phase. Total open esophagectomy was preferred by 21% of the respondents. Total minimally invasive esophagectomy was significantly more often performed in high-volume centers compared with non-high-volume centers (*P* = 0.002). Robotic assistance was used in 13% during the thoracic phase and 6% during the abdominal phase. Minimally invasive transthoracic esophagectomy has become the preferred approach for esophagectomy. Although 21% of the surgeons prefer an open approach, 26% of the surgeons perform a hybrid procedure which may reflect further transition towards the use of total minimally invasive esophagectomy.

## INTRODUCTION

Esophageal cancer is one of the most common cancers worldwide, ranking seventh in incidence.^1^ The preferred treatment at curative intent is neoadjuvant (or perioperative) therapy followed by esophagectomy, achieving a 5-year survival rate of 40–50%.^2^^,^^3^ Esophageal surgery is a highly complex procedure and is not yet standardized as many technical details can vary between surgeons, centers and countries. To gain insight in worldwide practice and trends of esophageal and gastroesophageal junction cancer surgery, an international survey was previously conducted in 2007 and 2014.^4–6^

The previous surveys demonstrated that open esophagectomy was the preferred approach worldwide but minimally invasive esophagectomy (MIE) increased in popularity from 2007 to 2014. In addition, an increase in high-volume centers was observed. The survey also demonstrated the controversy in treatment strategy for patients with Siewert type 2 tumors as both esophagectomy and gastrectomy were frequently performed.

From 2014 onwards, surgical techniques have continued to evolve and randomized trials have shown the superiority of the (robot-assisted) (RA) MIE approach over open surgery.^7^^,^^8^There is an increased interest in robotic surgery and the use of fluorescence techniques to determine the location of the esophagogastric anastomosis.^9^^,^^10^ A new survey was therefore sent out in 2021 aiming to update the current practice of esophageal and gastroesophageal junction cancer surgery worldwide. Furthermore, as the items were comparable to the previous surveys of 2007 and 2014, trends of a 7-yearly period could be analyzed.

## METHODS

An English online survey was developed based on questions of the survey in 2007 and 2014 (Supplementary 1). Several refinements were made to the survey to include current topics of interest. Topics that were already clarified in the previous surveys were removed, including the preferred reconstruction technique (95% gastric conduit) and the preferred type of thoracotomy (93% right sided). New items on the survey were questions on robotic esophagectomy, fluorescent techniques, details on postoperative feeding and question about postoperative surveillance. The final survey included questions about demographics, surgical approach and details and postoperative surveillance. Based on the privacy principles, we have no information about the respondents’ names nor the hospital they work in. High-volume centers were defined if > 51 esophagectomies were performed yearly and non-high-volume centers were defined if < 50 esophagectomies were performed yearly, based on literature.^11–14^

**Fig. 1:**
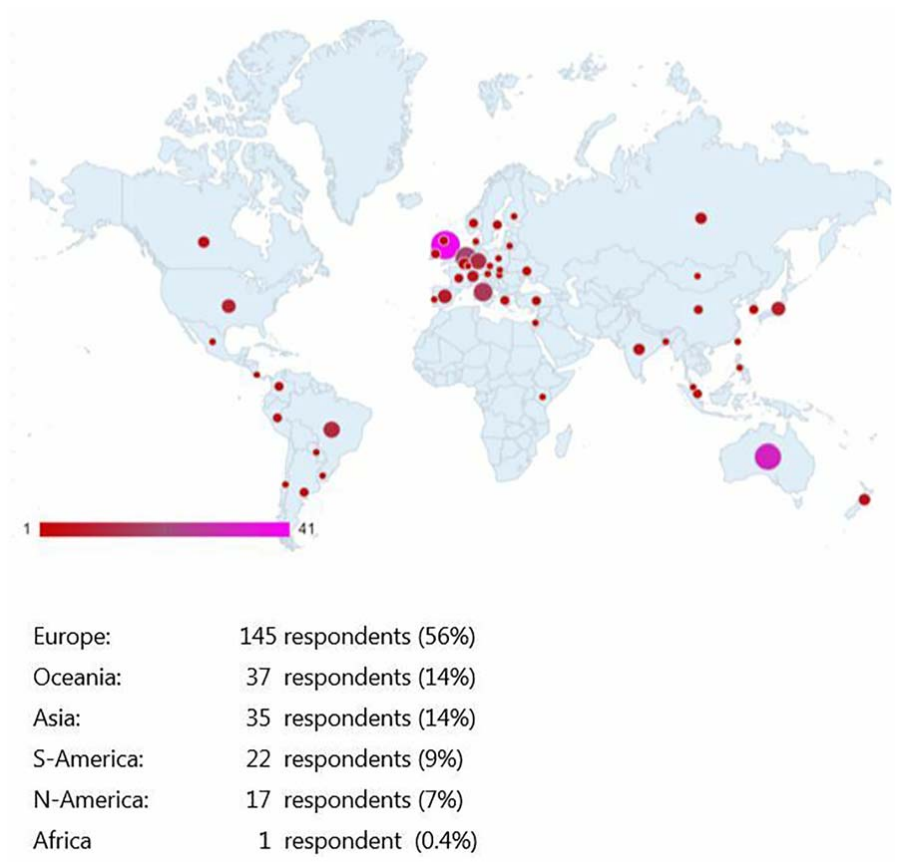
Distribution of respondents per participating country.

The survey was sent out to 414 members of the International Society for Diseases of the Esophagus (ISDE), 20 members of the World Organization for Specialized Studies on Disease of the Esophagus (OESO), 1268 members of the International Gastric Cancer Association (IGCA) and 400 members of Association of Upper Gastrointestinal Surgery of Great Britain and Ireland (AUGIS). The members of the IGCA only received the questions on gastroesophageal junction cancer. All associations were approached to send out the survey to their members. In addition, the survey was forwarded to Dutch gastroesophageal surgeons via the network of the investigators (JR, RvH). The invitations for participation were sent in April 2021 (IGCA), May 2021 (ISDE, OESO, European Society for Diseases of the Esophagus [ESDE]/IGCA, Dutch gastroesophageal surgeons network), July 2021 (AUGIS) and in November 2021 (ISDE, second time). The survey was also distributed at the ESDE congress in Milano (18–20 November 2021) by showing a QR-code after oral presentations. All replies were checked by hand to identify duplicates by checking email-addresses. In case of doubles, only the most recent response was included. The survey was closed on 31 December 2021. Outcomes were reported as a number with percentage. Descriptive analyses were performed for all outcomes except for the comparison between high-volume centers and non-high-volume centers. Comparison analyses were performed by a Chi-square test. A *P*-value below 0.05 was considered as a statistically significant difference.

## RESULTS

### Demographics

The survey was completed by 299 surgeons of which 54 respondents from the IGCA and 206 from the other organizations. After removal of 39 duplicates, 260 respondents were included in this study. The response rate was 12% (260/2102). The respondents represented 52 different countries and 6 continents (Fig. 1). The majority of the respondents indicated that they worked in a university hospital (77%), followed by a regional (17%) and local hospital (6%).

#### Hospital volume

**Fig. 2:**
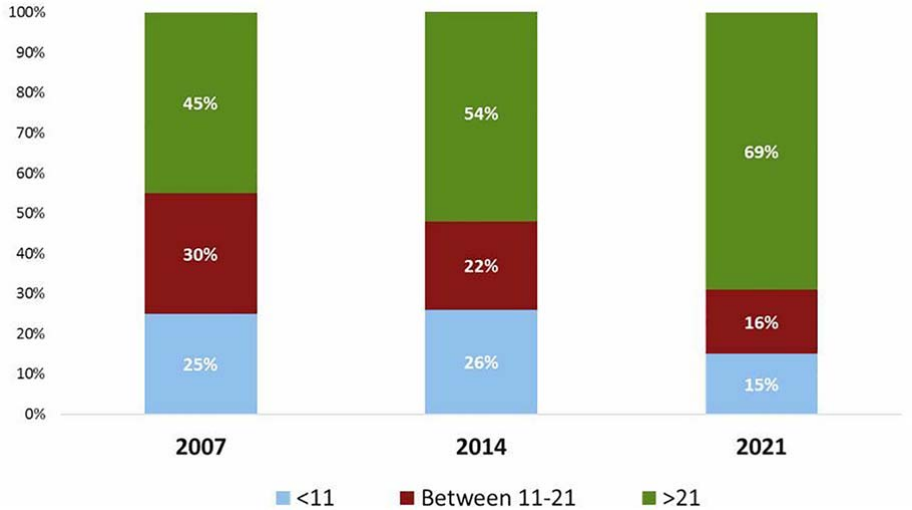
Number of esophagectomies performed in the hospital annually.

**Fig. 3:**
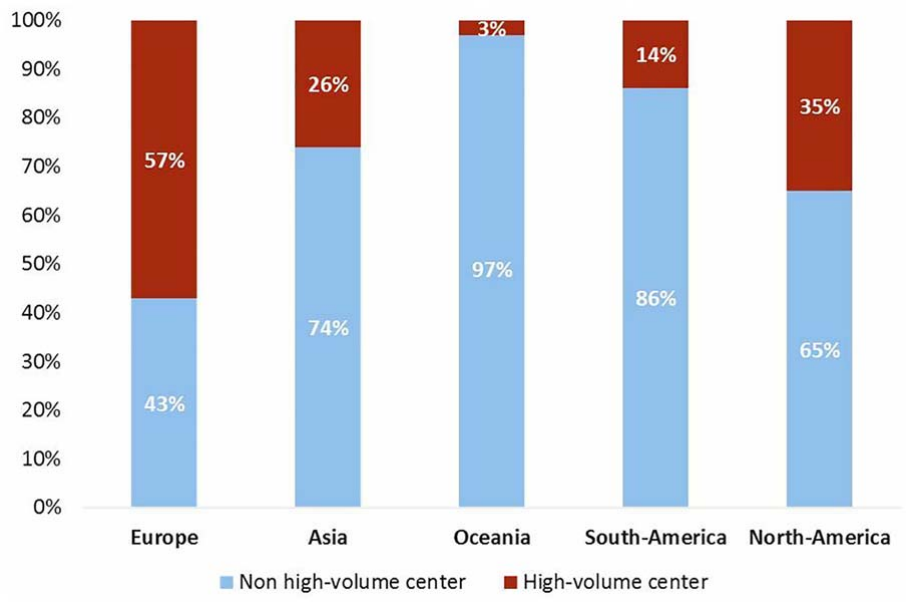
High-volume centers per continent. High-volume centers is defined as >51 esophagectomies annually and non-high-volume center as <50 esophagectomies annually.

To compare hospital volume to the surveys in 2007 and 2014, the number of yearly performed esophagectomies was grouped into <11 esophagectomies, 11–21 esophagectomies and > 21 esophagectomies. As demonstrated in Figure 2, the number of >21 esophagectomies performed yearly increased from 45% in 2007 to 54% in 2014 and 69% in 2021. In 2021, 39% of the respondents worked in a hospital that performed >51 esophagectomies per year (high-volume hospital). The high-volume respondents mainly originated from Europe as the majority of the respondents from the other continents worked in a non-high-volume hospital (Fig. 3).

### Esophageal cancer surgery

Details on esophageal cancer surgery are presented in Table 1.

**Table 1:** Details on esophageal cancer surgery

	**%**
**Preferred approach** Transthoracic Transhiatal	96% 4%
**Approach abdominal phase** Laparoscopy Laparotomy Robot-assisted	67% 28% 6%
**Approach thoracic phase** Thoracoscopy Thoracotomy Robot-assisted Not applicable (transhiatal)	45% 39% 13% 3%
**Approach** Total open Hybrid – thoracoscopic Hybrid – laparoscopic Total MIE	21% 7% 19% 53%
**Width gastric conduit** ≥ 5 centimeters 4 centimeters 3 centimeters 2 centimeters	22% 51% 25% 2%
**Positioning reconstruction** Esophageal bed Retrosternal	98% 2%
**Intrathoracic anastomosis** Proximal tumors Mid tumors Distal tumors	6% 56% 85%
**Cervical anastomotic technique** Hand-sewn Linear stapled Circular stapled	70% 25% 4%
**Intrathoracic anastomotic technique** Hand-sewn Linear stapled Circular stapled	23% 24% 52%
**Routinely a jejunal feeding tube**	55%
**Routinely a pyloromyotomy**	31%
**Use of indocyanine green for the location of the anastomosis**	29%
**Restart oral feeding after esophagectomy** Within 1 day Within 1 week After 2 weeks	12% 85% 2%

#### Approach

**Fig. 4:**
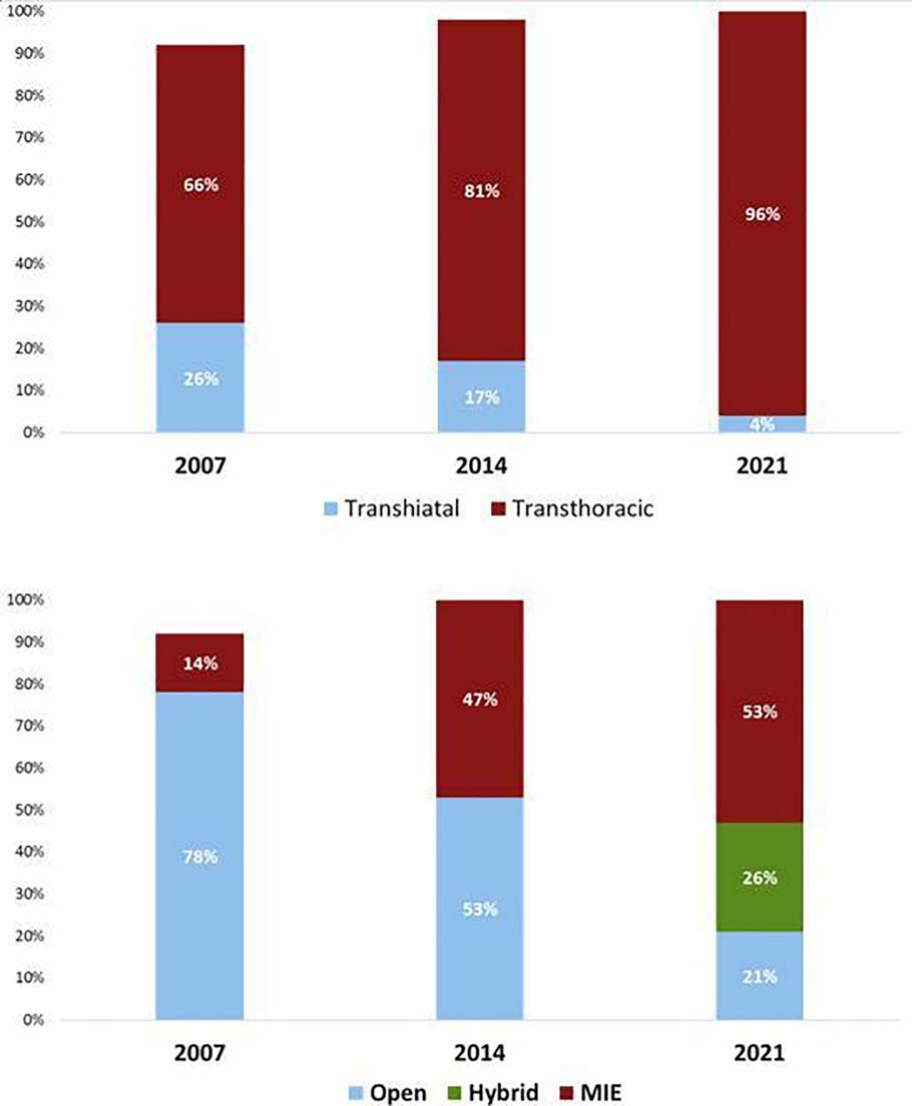
Preferred approach to perform an esophagectomy over the years.

In 2021, a transthoracic esophagectomy was the preferred approach for 96% of the respondents, whereas a transhiatal approach was preferred by the other 4%. When comparing these results to 2007 and 2014, an increase towards a transthoracic procedure as the preferred technique is observed from 66% in 2007 to 81% in 2014 (Fig. 4). In 2021, for the abdominal phase of esophagectomy, the majority of the surgeons preferred laparoscopy (67%) followed by laparotomy (28%) and robotic assistance (6%). In 2021, the preferred technique for the thoracic phase was thoracoscopic (45%) followed by thoracotomy (39%) and robotic assistance (13%). For surgeons who performed a minimally invasive thoracic phase, the patient was positioned in semiprone (40%), prone (41%) or left lateral (19%).

**Fig. 5:**
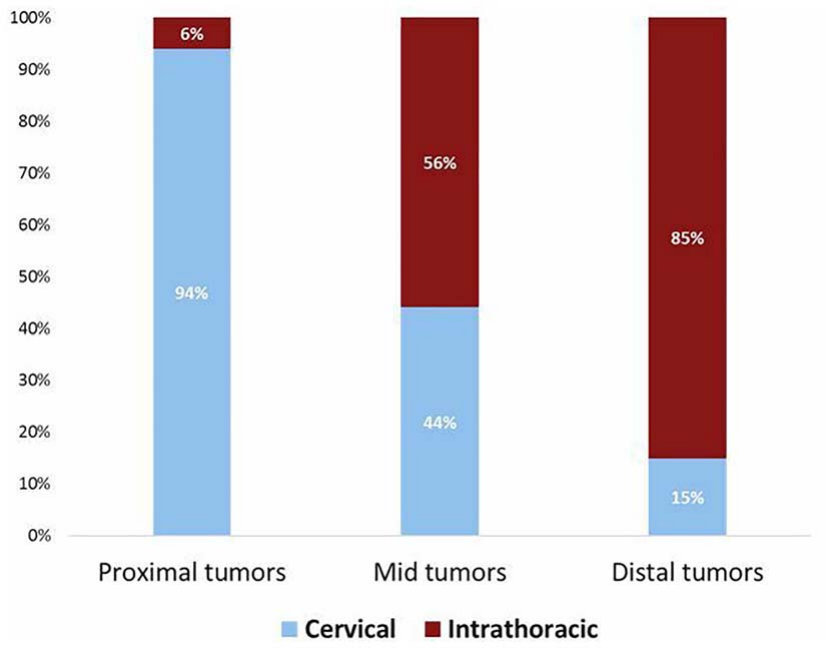
Preferred location of the anastomosis according to the level of the tumor.

Overall, 53% of the surgeons preferred a total MIE, 21% a total open esophagectomy and 26% a hybrid procedure (7% a minimally invasive thoracic phase combined with laparotomy and 19% a minimally invasive abdominal phase combined with thoracotomy). The preferred location for the anastomosis in relative to the tumor location is shown in Fig. 5.

#### High-volume centers

Differences on surgical care for esophageal cancer were compared between high-volume centers (>51 yearly esophagectomies) and non-high-volume centers (<50 yearly esophagectomies) and are demonstrated in Table 2. The approach for the abdominal phase during esophagectomy significantly differed between high-volume and non-high-volume centers (*P* = 0.012) in which laparoscopy (71 vs. 63%) and a robot-assisted approach was more often performed (10 vs. 3%) in high-volume centers. A total MIE procedure was preferred more in high-volume centers (65 vs. 43%) and a hybrid procedure more often in non-high-volume centers (34 vs.16%) which was statistically significant (*P* = 0.002). High-volume centers more often created an intrathoracic anastomoses than a cervical anastomoses for proximal (*P* = 0.003) and mid (*P* = 0.014) esophageal tumors compared with non-high-volume centers. The routine placement of a feeding jejunostomy and the routine use of indocyanine green (ICG) were similar between both groups. Non-high-volume centers more often performed a routine pyloromyotomy (37 vs. 23%, *P* = 0.057).

**Table 2:** Details on esophageal and gastroesophageal junction cancer surgery for high-volume centers and non-high-volume centers

	**Non-high volume** <50 esophagectomies	**High volume** >51 esophagectomies	***P*-value**
**Esophageal cancer surgery**			
**Approach abdominal phase** Laparoscopy Laparotomy Robot-assisted	63% 34% 3%	71% 20% 10%	**0.012**
**Approach thoracic phase** Thoracoscopy Thoracotomy Robot-assisted	45% 43% 9%	46% 34% 17%	0.224
**Approach** Total open Hybrid Total MIE	23% 34% 43%	19% 16% 65%	**0.002**
**Intrathoracic anastomosis** Proximal tumors Mid tumors Distal tumors	1% 47% 81%	12% 66% 90%	**0.003** **0.014** 0.138
**Cervical anastomotic technique** Hand-sewn Linear stapled Circular stapled	77% 21% 2%	62% 30% 8%	**0.028**
**Intrathoracic anastomotic technique** Hand-sewn Linear stapled Circular stapled	28% 26% 46%	17% 22% 61%	0.075
**Jejunal feeding tube**	53%	58%	0.389
**Pyloromyotomy**	37%	23%	0.057
**Indocyanine green**	28%	29%	0.658
**Gastroesophageal junction cancer surgery**			
**Approach Siewert type I** Transthoracic esophagectomy Transhiatal esophagectomy	88% 12%	98% 2%	**0.004**
**Approach Siewert type II** Transthoracic esophagectomy Transhiatal esophagectomy Extended gastrectomy Proximal gastrectomy	65% 14% 16% 4%	88% 5% 7% 0%	**0.000**
**Approach Siewert type III** Transthoracic esophagectomy Transhiatal esophagectomy Extended gastrectomy Proximal gastrectomy	4% 6% 82% 7%	11% 2% 84% 3%	**0.043**

The bold values indicate that the *p* value is <0.05.

### Gastroesophageal junction cancer

#### Diagnosis and classification

The most important diagnostic instrument to determine the location of the gastroesophageal junction tumor was an esophagogastroscopy according to 69% of the respondents followed by a PET-scan in 9%, computed tomography-scan in 8%, diagnostic laparoscopy in 8% and endoscopic ultrasound (EUS) in 5%. To preoperatively determine the surgical strategy with regards to the primary tumor of gastroesophageal junction cancers, 73% of the surgeons used both the Siewert classification and the TNM classification, whereas 19% only used the Siewert classification and 8% only the TNM classification. In 2014, both classifications were used by 45% of the respondents, the Siewert classification in 39% and the TNM classification only in 16%.

#### Surgical treatment

**Fig. 6:**
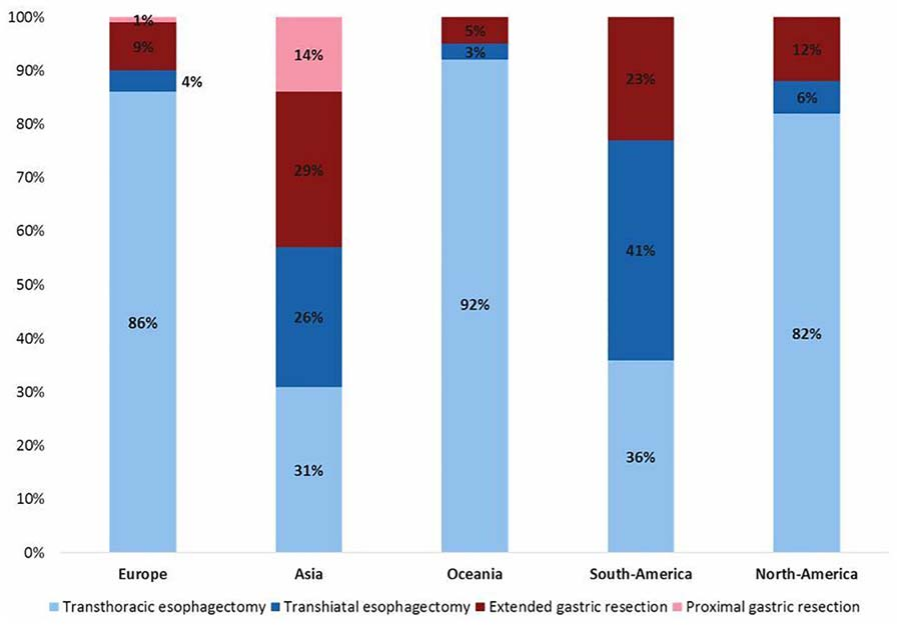
Preferred surgical approach for gastroesophageal junction Siewert type 2 cancer in 2021 per continent.

The type of surgical treatment depended on the tumor location according to the Siewert classification. All surgeons performed an esophagectomy for Siewert type I carcinomas (92% transthoracic, 8% transhiatal). For Siewert type 3 carcinomas, an extended gastrectomy was preferred by 83% of the surgeons, an esophagectomy by 11% and a proximal gastrectomy by 5%. For Siewert type 2 carcinomas, the preferred treatment differed between continents (Fig. 6). In Europe, the majority of the respondents preferred an esophagectomy (90%) as a gastrectomy was only preferred by 10% whereas in Asia, an esophagectomy was preferred by 57% and a gastrectomy by 43% of the respondents. In Oceania, South-America and North-America, the esophagectomy was the preferred approach over a gastrectomy. When comparing these results to 2014, an increase in overall preference for an esophagectomy for Siewert type 2 cancer was observed as this was overall only 28% in 2014.

In 44% of the surgeons, the planned surgical strategy for gastroesophageal junction (GEJ) tumors changed during surgery between 0 and 5% of the cases. The planned surgical strategy changed between 5 and 10% of the cases in 32% of the respondents and between 10 and 20% in 17%. In 8% of the surgeons, the planned surgical strategy changed during > 20% of the surgeries. When performing an esophagectomy for Siewert type 2 cancer, most surgeons performed a combined abdominal and thoracic lymphadenectomy (59%) (Supplementary 2). In 22% of the surgeons, only an abdominal combined with a lower thoracic lymphadenectomy was performed and in 17% an abdominal, thoracic and paratracheal lymphadenectomy.

### Follow-up

**Fig. 7:**
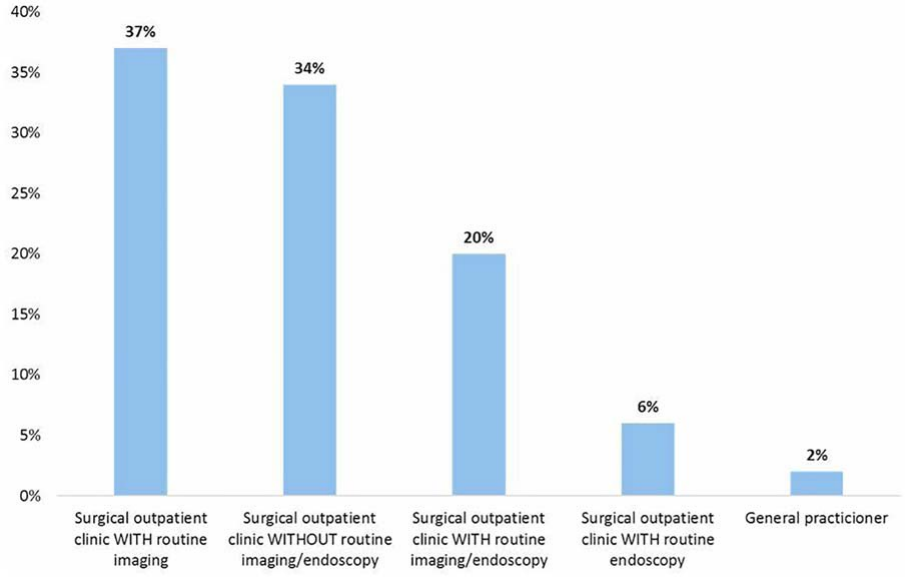
Preferences of follow-up during the first year after esophagectomy.

Figure 7 represents the type of postoperative surveillance that is used in the first year after esophagectomy. Most surgeons used regular visits to the surgical department with routine imaging (37%) followed by regular visits without routine imaging or endoscopy (34%), regular visits with routine imagine combined with endoscopy (20%) and regular visits with endoscopy only (6%). In the remaining 3%, the follow-up was at the general practitioner.

The pathological TNM stage influenced the decision on which postoperative surveillance to use in 45% of the respondents.

## DISCUSSION

This study demonstrates the current worldwide practice in esophageal and gastroesophageal junction cancer surgery. In addition, trends were identified by comparing the results of this survey to previous similar surveys conducted in 2007 and 2014. MIE has become the preferred approach instead of open esophagectomy as a total MIE was preferred by 53%, a hybrid procedure by 26% (7% a minimally invasive thoracic phase combined with laparotomy and 19% a minimally invasive abdominal phase combined with thoracotomy) and a total open esophagectomy by 21% of the surgeons. Total MIE was significantly more often performed in high-volume centers compared with non-high-volume centers, whereas a hybrid procedure was more often performed in non-high-volume centers (*P* = 0.002). This is in line with a recent study on trends in esophagectomy including 39 high-volume centers and evaluated the data of 6022 esophagectomies. They showed that 53% of the procedures consisted of a MIE.^15^ Although minimally invasive transthoracic esophagectomy continues to gain acceptance, the rise in application might have reached a plateau over the past 7 years. A hybrid approach is often used as an alternative approach for total MIE and may reflect a transition phase from open surgery to MIE.

This is the first time this survey included questions regarding the use of robot-assisted surgery. A robot-assisted thoracic phase was the preferred approach in 13% of the surgeons and a robot-assisted abdominal phase in 6%. Robotic surgery has several, mainly technical, advantages over conventional minimally invasive surgery and might improve the lymph node dissection.^16^^,^^17^ However, whether robotic surgery improves patient outcomes compared with conventional minimally invasive surgery is not yet clarified. One randomized controlled multicenter trial in China with 362 patients compared robotic esophagectomy to MIE.^18^ No differences in postoperative complications were observed. However, robot-assisted esophagectomy achieved a shorted operation time and improved lymph node yield compared with MIE. Currently, two other randomized controlled trials are underway (ROBOT-II trial and REVATE trial) comparing robotic esophagectomy to MIE with lymph node yield and recurrent nerve palsy as primary outcome.^19^^,^^20^ The outcomes of these trials will influence future trends in the use of robot-assisted esophagectomy.

In line with the increase in MIE, a transthoracic esophagectomy has become the dominant approach as 96% of the respondents preferred a transthoracic approach over a transhiatal esophagectomy, whereas this was 66% in 2007 and 81% in 2014. Several factors likely have contributed to this transition. First, the simultaneously rise of MIE. Surgeons might initially have avoided an open transthoracic esophagectomy as it is an invasive procedure associated with significant morbidity compared with a transhiatal esophagectomy.^21^ However, with the rise of MIE, a transthoracic procedure has become less invasive and postoperative outcomes improved.^22^^,^^23^ Second, recent evidence demonstrated that an intrathoracic anastomosis is superior over a cervical anastomosis in terms of anastomotic leakage and functional outcomes which is only possible during a transthoracic approach^24^^,^^25^. Last, the lymphadenectomy during transhiatal esophagectomy is inferior to transthoracic esophagectomy.^21^^,^^26^ The location for the esophagogastric anastomosis depends on several factors including the extension of the radiation field and tumor level. In general, proximal tumors require a cervical anastomosis from an oncological point of view, whereas for distal tumors an intrathoracic anastomosis is more appropriate. For tumors located in the mid esophagus, the preferred location for the anastomosis was controversial as 44% preferred a cervical anastomosis and 56% an intrathoracic anastomosis. Although intrathoracic anastomoses are associated with better outcomes as mentioned before, they are also demonstrated to be a risk factor for irradical resection marges.^27^ Therefore, an intrathoracic anastomosis should be reserved for distal esophageal cancer.

In line with the previously conducted surveys, esophagectomy was the preferred approach for Siewert type 1 carcinomas and gastrectomy for Siewert type 3 carcinomas. For Siewert type 2 carcinomas, the results differ from the previous survey as the majority of the surgeons preferred an esophagectomy in 2021 in contrast to a gastrectomy in 2014 in all continents. As demonstrated previously, gastric surgeons prefer a gastrectomy over esophagectomy for Siewert type 2 cancer. It could be possible that this time relatively less gastric cancer surgeons responded compared with esophageal cancer surgeons. Another reason could be that more surgeons from high-volume hospitals have completed the survey who seem to prefer an esophagectomy slightly more often compared with surgeons working in non-high-volume hospitals.

The last part of the survey consisted of questions about surveillance after esophagectomy. The most common surveillance strategies were visits at the surgical outpatient clinic with routine imaging (37%) and visits at the surgical outpatient clinic without routine imagine and/or endoscopy (34%). Recently, a study on postoperative surveillance demonstrated similar results. In that study, also 37% of the centers used postoperative surveillance with annual imaging.^28^ Postoperative surveillance with routine imaging has shown to improve survival in patients with early stage disease. These data establish the need for studies to conclude on the most optimal postoperative surveillance strategy.

A strength of this survey is the repeating character of the questions which makes it possible to compare results to 2007 and 2014 and therewith identifying trends. However, not all the questions are identical to the previous surveys because some of the questions were considered outdated or were added as they have gained increased attention during the last year. Several limitations apply to this survey as well. First, although a high number of surgeons from all over the world responded, the relative limited response rate might have induced bias. For example, high-volume centers with special interest in esophageal surgery may be more likely to respond, explaining in part the high percentage (39%) of respondents performing > 50 esophagectomies a year. This could have led to an overestimation of minimally invasive esophagectomies. Another limitation is that more than half of the respondents came from Europe which could have influenced the results. Last, the proportion of gastric/esophageal surgeons and subspecialties (upper-GI surgeon, general surgeon, thoracic surgeon) was unknown which would have been useful information to interpret the results and trends.

In conclusion, this survey reflects the worldwide preferences of surgery for esophageal and gastroesophageal junction cancer. Compared with 2007 and 2014, MIE has become the preferred approach over the open approach.

## ACKNOWDLEDGEMENTS

The authors would like to thank the ISDE, OESO, IGCA and AUGIS for sending out the survey. The authors highly appreciate the cooperation of all the respondents.

## Supplementary Material

Supplementary_1_survey_doac099

Supplementary_2_doac099
