# Supplementary material for: Trends in surgical techniques for the treatment of esophageal and gastroesophageal junction cancer: the 2022 update
Source: Dis Esophagus. 2023 Jan 12;36(7):doac099. doi: 10.1093/dote/doac099 (PMC10317002; doi:10.1093/dote/doac099)
Supplement: Supplementary_1_survey_doac099 [file supplementary_1_survey_doac099.pdf]

Survey esophageal and gastroesophageal junction surgery 2021

The aim of this survey is to evaluate current surgical techniques that are performed worldwide. We will compare the results to similar surveys we have performed previously, in order to identify the changes and trends in surgical techniques (Haverkamp et al. Dis Esoph 2017;30: and Boone et al. Dis Esoph 2009;22(3):195-202).
The survey will take approximately 5 minutes.

Best regards,

Prof.dr. Richard van Hillegersberg
Department of Surgery, G04.228
University Medical Center Utrecht
Heidelberglaan 100, 3584CX Utrecht, the Netherlands
+31 (0)88-7558074
F: +31 (0)30-2541944
r.vanhillegersberg@umcutrecht.nl

Demographic data

1. What is your email address? Optional for feedback to you. Data will be analyzed anonymously

____

2. What country do you live in?

____

3. What kind of clinic do you work in?

Markeer slechts één ovaal.

- ⬭ University hospital
- ⬭ Regional hospital
- ⬭ Local hospital

4. How many esophagectomies are performed in your HOSPITAL each year?

Markeer slechts één ovaal.

- 0
- <11
- 11-20
- 21-30
- 31-40
- 41-50
- 51-60
- >60

Gastroesophageal Junction (GEJ) Tumors

The following questions are about GASTROESOPHAGEAL JUNCTION tumors in specific.

5. Please rank the following diagnostics in order of importance in determining the LOCATION of gastroesophageal junction tumors. (1= most important, 5=least important)

Each number could only be chosen once

Markeer slechts één ovaal per rij.

	1	2	3	4	5
**CT-scan**					
**Esophagogastroscopy**					
**EUS**					
**PET-scan**					
**Diagnostic laparoscopy**					

Siewert classification of GEJ tumors. Type I = green (1–5 cm proximal from esophagogastric junction). Type II = blue (1 cm proximal to 2 cm distal from esophagogastric junction). Type III = purple (2–5 cm distal from esophagogastric junction).

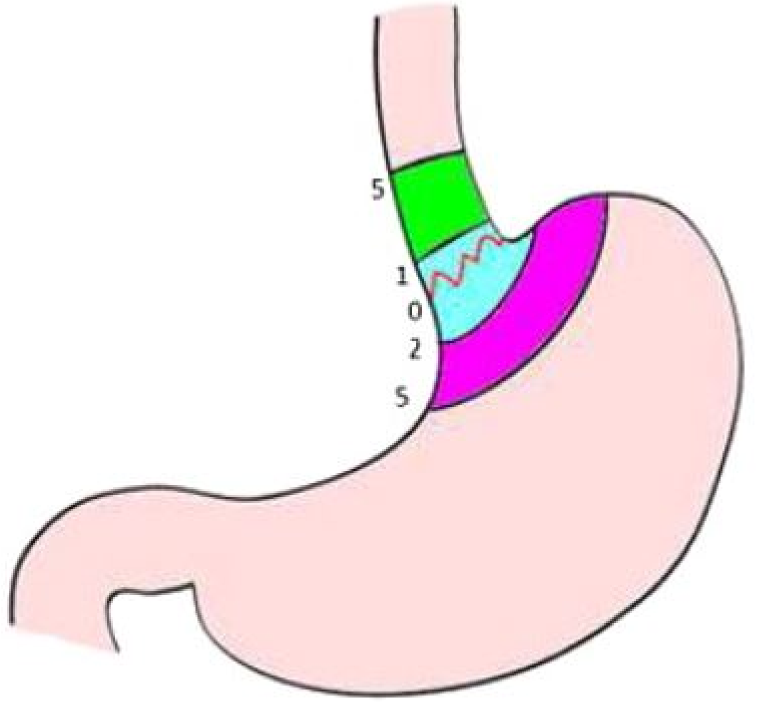

L. Haverkamp et al. *Systematic review of the surgical strategies of adenocarcinomas of the gastroesophageal junction.* Surg Onc 2014. Volume 23 p222-228

6. What is the preferred surgical approach for Siewert type I, type II and type III carcinomas?

Markeer slechts één ovaal per rij.

	Transthoracic esophagectomy	Transhiatal esophagectomy	Extended gastric resection	Proximal gastric resection
**Siewert type I**	◯	◯	◯	◯
**Siewert type II**	◯	◯	◯	◯
**Siewert type III**	◯	◯	◯	◯

7. Do you preferably make use of the Siewert classification, TNM classification, or both in determining preoperative surgical strategy for gastroesophageal junction tumors?

Markeer slechts één ovaal.

- Siewert classification
- TNM classification
- Both

8. How often do you change the planned surgical strategy during surgery for gastroesophageal junction tumors?

Markeer slechts één ovaal.

- 0-5%
- 5-10%
- 10-20%
- 20-30%
- 30-40%
- 40-50%
- 50-60%
- 60-70%
- 70-80%
- 80-90%
- 90-100%

Lymph node stations

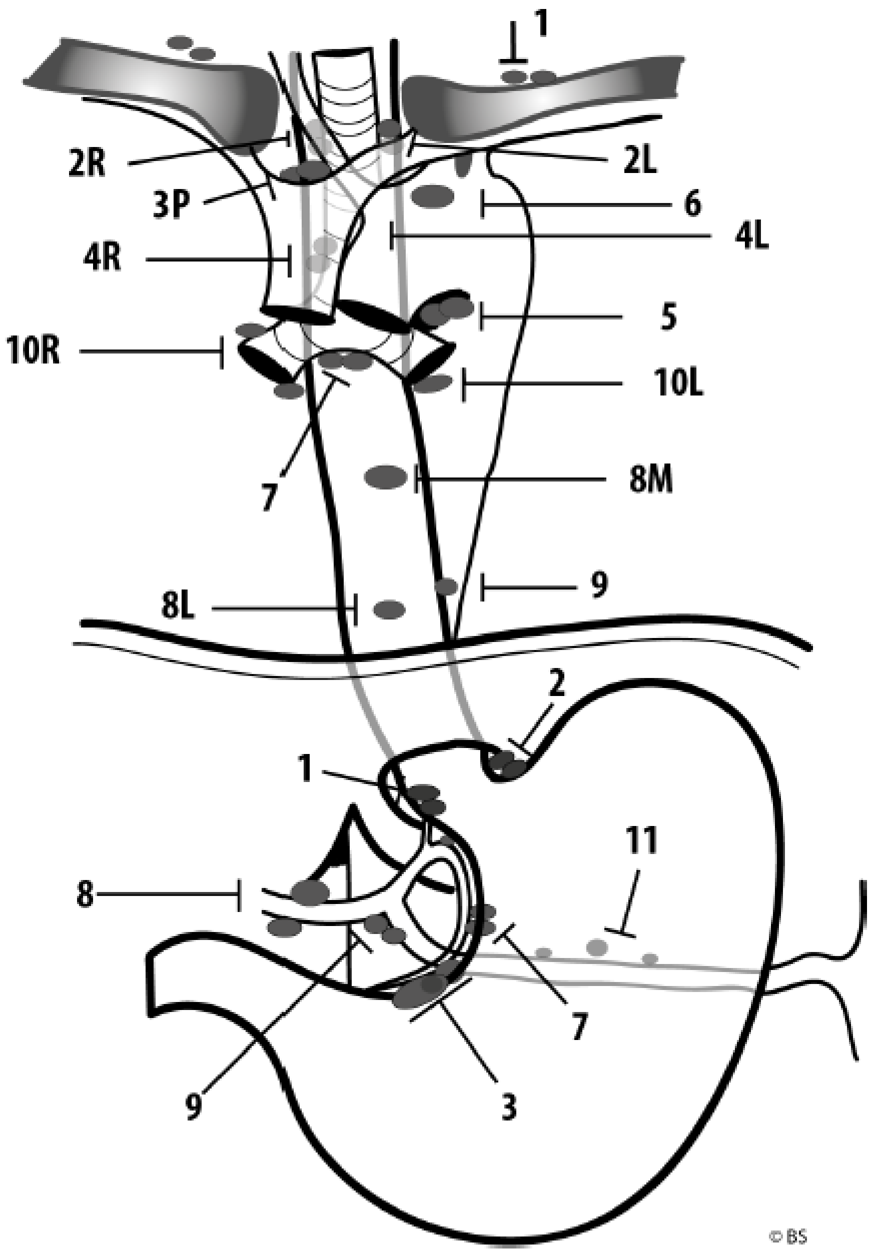

9. If you perform an esophageal resection for a SIEWERT type II gastroesophageal junction tumor, what is the preferred extent of your lymph node dissection?

Markeer slechts één ovaal.

- Abdominal (station 3,7,8,9)
- Abdominal and lower thoracic (abdomen station 3,7,8,9 and thoracic station 8L and 9)
- Abdominal and thoracic (abdominal station 3,7,8,9 and thoracic station 7,8,9,10)
- Abdominal, thoracic and paratracheal (abdominal station 3,7,8,9 and thoracic station 7,8,9,10 and 4,5)
- Abdominal, thoracic and cervical

Esophageal cancer

The following questions are about ESOPHAGEAL cancer in specific.

10. What is the prefered surgical approach for esophageal cancer?

Markeer slechts één ovaal.

- Transhiatal
- Transthoracic

11. What is the prefered approach for the ABDOMINAL phase?

Markeer slechts één ovaal.

- Laparotomy
- Laparoscopy
- Robot-assisted

12. What is the prefered approach for the THORACIC phase?

Markeer slechts één ovaal.

- Thoracotomy
- Thoracoscopic
- Robot-assisted
- Not applicable, I prefer transhiatal

13. How do you position the patient during the THORACIC phase of esophagectomy?

Markeer slechts één ovaal.

- Prone
- Semiprone
- Left lateral

14. If you construct a gastric conduit after esophagectomy, what is the estimated width of the constructed gastric conduit?

Markeer slechts één ovaal.

- 2 cm
- 3 cm
- 4 cm
- 5 cm
- >5 cm

15. Where do you preferably position the reconstruction after esophagectomy?

Markeer slechts één ovaal.

- Esophageal bed
- Retrosternal
- Subcutaneous

16. What is the preferred location of the anastomosis after esophagectomy for:

Markeer slechts één ovaal per rij.

	Cervical	Intrathoracic
**Proximal tumors**	⬭	⬭
**Mid tumors**	⬭	⬭
**Distal tumors**	⬭	⬭

17. How do you preferably construct the CERVICAL anastomosis after esophagectomy?

Markeer slechts één ovaal.

- ⬭ Mechanical, linear stapler
- ⬭ Mechanical, circular stapler
- ⬭ Hand-sewn 1-layer
- ⬭ Hand-sewn 1-layer with a few tension releasing stiches
- ⬭ Hand-sewn 2-layer

18. How do you preferably construct the INTRATHORACIC anastomosis after esophagectomy?

Markeer slechts één ovaal.

- ⬭ Mechanical, linear stapler
- ⬭ Mechanical, circular stapler
- ⬭ Hand-sewn 1-layer
- ⬭ Hand-sewn 1-layer with a few tension releasing stiches
- ⬭ Hand-sewn 2-layer

19. Do you use intraoperative fluorescence techniques to guide the location for the anastomotic site?

Markeer slechts één ovaal.

- Yes
- No

20. Do you routinely perform a pyloromyotomy?

Markeer slechts één ovaal.

- Yes
- No

21. Do you routinely place a jejunostomy feeding tube during esophagectomy?

Markeer slechts één ovaal.

- Yes
- No

22. On what postoperative day do you resume oral feeding?

Markeer slechts één ovaal.

- Within 1 day
- Within 1 week
- After 2 weeks

Follow-up

23. What type of postoperative surveillance do you use in the first years after esophagectomy?

Vink alle toepasselijke opties aan.

- [ ] No regular visits
- [ ] Regular visits to the general practitioner
- [ ] Regular visits to the surgical outpatient clinic WITHOUT routine imaging or endoscopy
- [ ] Regular visits to the surgical outpatient clinic WITH routine imaging
- [ ] Regular visits to the surgical outpatient clinic WITH routine endoscopy

24. Does the pathological TNM stage influence your decision on which postoperative surveillance to use?

Markeer slechts één ovaal.

- Yes
- No

Deze content is niet gemaakt of goedgekeurd door Google.

Google Formulieren
