# Supplementary material for: Trends in surgical techniques for the treatment of esophageal and gastroesophageal junction cancer: the 2022 update
Source: Dis Esophagus. 2023 Jan 12;36(7):doac099. doi: 10.1093/dote/doac099 (PMC10317002; doi:10.1093/dote/doac099)

**Supplementary 1**

**Figure 1. Lymphadenectomy during esophagectomy for a SIEWERT type II tumors.**


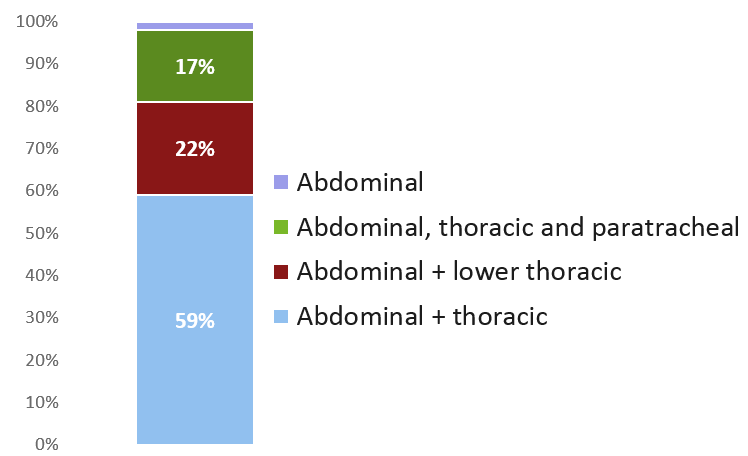


**Figure 2. Anastomotic techniques for intrathoracic and cervical anastomosis.**


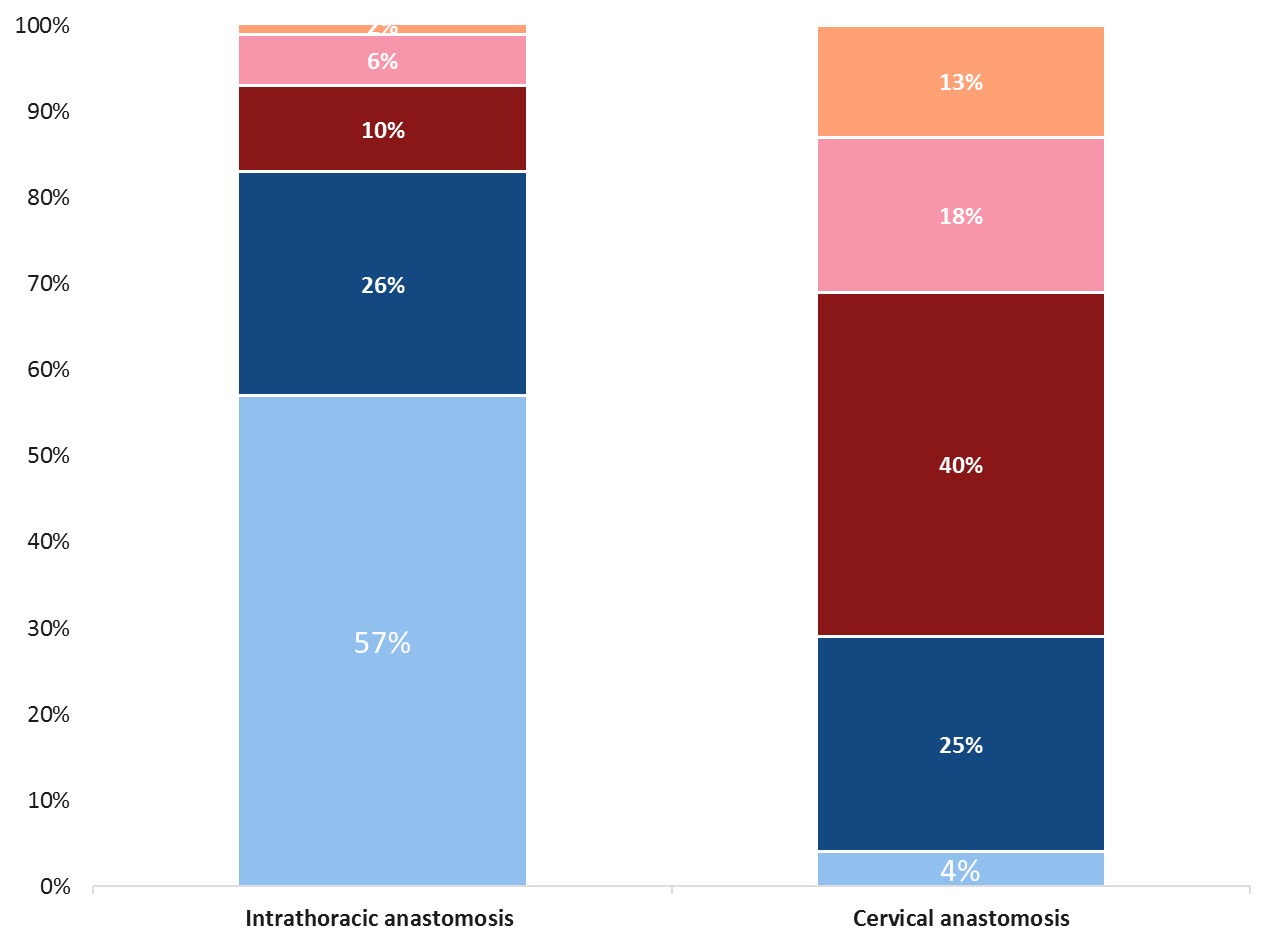


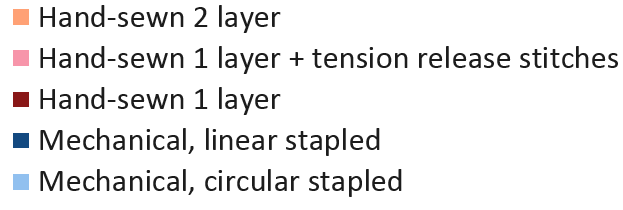

Supplement: Supplementary_2_doac099 [file supplementary_2_doac099.docx]
